# Transcriptome and metabolome analyses reveal anthocyanins pathways associated with fruit color changes in plum (*Prunus salicina* Lindl.)

**DOI:** 10.7717/peerj.14413

**Published:** 2022-12-13

**Authors:** Lei Chen, Xuesong Wang, Long Cui, Yuebo Li, Yinghai Liang, Shanshan Wang, Yubo Chen, Lan Zhou, Yanbo Zhang, Feng Li

**Affiliations:** 1 Institute of Pomology, Jilin Academy of Agricultural Sciences, Changchun, Jilin Province, China; 2 Academy of Agricultural Sciences of Yanbian, Longjing, Jilin Province, China

**Keywords:** Plum, Fruit color, Transcriptome, Metabolome, Anthocyanins

## Abstract

Plum (*Prunus salicina* Lindl.) is one of the most widely cultivated and important fruit trees in temperate and cold regions. Fruit color is a significant trait relating to fruit quality in plum. However, its development mechanism has not been studied from the aspects of transcriptional regulation and metabolomic progress. To reveal the mechanism of fruit color developments in plums, we selected the fruits of two plum cultivars, ‘*Changli84*’ (Ch84, red fruit) and ‘*Dahuangganhe*’ (D, yellow fruit) as plant materials for transcriptome sequencing and metabolomic analysis were performed. Based on the data of transcriptome and metabolome at three fruit developmental stages, young fruit stage, color-change stage, and maturation stage, we identified 2,492 differentially expressed genes (DEGs) and 54 differential metabolites (DMs). The KEGG analysis indicated that “Flavonoid biosynthesis” was significantly enriched during three fruit development stages. Some DEGs in the “Flavonoid biosynthesis” pathway, had opposite trends between Ch84 and D, including chalcone synthase (*CHS*), dihydroflavonol 4-reductase (*DFR*) and flavonol synthase (*FLS*). Also, the genes encoding MYB–bHLH–WD (MBW) protein complexes, especially *MYBs* and *bHLHs*, showed a close relationship with plum fruit color. In the current study, DMs like procyanidin B1, cyanidin 3-glucoside, and cyanidin-3-O-alpha-arabinopyranoside were key pigments (or precursors), while the carotene and carotenoids did not show key relationships with fruit color. In conclusion, the anthocyanins dominate the color change of plum fruit. Carotenes and carotenoids might be related to the color of plum fruit, but do not play a dominate role.

## Introduction

Plum (*Prunus salicina* Lindl.) is a favorite fruit product of consumers for their delicious taste. Plum has a wide variety of uses and consumers typically prefer to eat fresh plums for their characteristic taste and rich nutrient substance (Roussos et al., 2016). There has been increasing consumer interest in the potential health benefits of dietary-derived phytochemicals such as anthocyanins (including anthocyanin derivatives), prevalent in *P. salicina*. There are many varieties of plums, which have different characteristics, such as maturity period, different taste, different fruit colors and so on. In this study, two plum cultivars with different fruit colors were selected to study the molecular mechanism related to fruit color formation.

The concentration of anthocyanins determines the color of fruits (Dinda et al., 2016; Kazimierski, Regula & Molska, 2019). Anthocyanins are responsible for the colors of numerous flowers, fruits, vegetables, and even cereals (Bittar et al., 2014), and can strongly contribute to food quality and appeal to consumers. Anthocyanins are the most abundant flavonoid compounds, which possess antioxidant and anti-inflammatory properties (Hertog et al., 1993; Prior & Wu, 2006). Anthocyanin synthesis is regulated by many external environmental factors, such as nutrient depletion, drought, pathogen infection, temperature, light, and genetic factors (Chalker-Scott, 1999). Different factors could cause content changes in anthocyanin abundance *via* different pathways. Genetic factors related to anthocyanin accumulation can be divided into two categories. One category is the biosynthetic genes that encode enzymes required for anthocyanin biosynthesis, such as follows: chalcone synthase (*CHS*), chalcone isomerase (*CHI*), flavanone-3-hydroxylase (*F3H*), dihydroflavonol 4-reductase (*DFR*), anthocyanidin synthase (*ANS*), and UDP glucose flavonoid 3-O-glucosyltransferase (*UF3GT*) (An et al., 2015; Patra et al., 2013; Hichri et al., 2011). Another category is regulatory genes that influence the intensity and pattern of anthocyanin biosynthetic genes, including two major classes of transcription factors, the myeloblastosis (*MYB*) and basic helix–loop–helix (*bHLH*) families, which, along with WD-repeat protein, form an MYB–bHLH–WDR transcription complex to regulate anthocyanin synthesis (Wu et al., 2019; Feller et al., 2011; Xu, Dubos & Lepiniec, 2015). Structural and regulatory genes had been identified and cloned in model plants such as *Arabidopsis*, maize (Zea mays), apple, *etc* (Holton & Cornish, 1995; Broun, 2005; Petroni & Tonelli, 2011; Zhai et al., 2014; Feng et al., 2012; Hu et al., 2019). It has been reported that anthocyanins are closely related to peel color, and pericarp has been reported to contain many phenolics, including anthocyanins, procyanidins, flavonoids, lignans, and sesquiterpenes (Ma et al., 2014b; Ma et al., 2014a; Zhang et al., 2004). Dayar et al. (2020) reported that the concentration of anthocyanins determines the color of fruits. The relationship between the biosynthesis of plum anthocyanins (including proanthocyanidins) and fruit color has been reported previously (Chen et al., 2022; Niu et al., 2017). However, the observed correlation needs careful verification and more evidences.

In this study, we selected two common plum cultivars ‘*Changli84*’ (Ch84) and ‘*Dahuangganhe*’(D) in Northeast China. The fruit color of Ch84 is red, while D is yellow. To reveal the mechanism of the difference in fruit color between the two varieties, transcriptome sequencing and metabolomic analysis were performed at three different developmental stages: young fruit stage, color-change stage, and maturation stage. We hypothesize that anthocyanin synthesis-related genes and anthocyanin metabolites play an important role in fruit color formation. The present study provided supporting data and theoretical basis for the study of formation of plum fruit color.

## Material and Methods

### Plant materials

All plum fruit pericarp tissues were collected from the experimental farm of Jilin Academy of Agricultural Sciences, Gongzhuling City, Jilin Province. Two varieties of plum trees, Changli 84 (Ch84) and ‘*Dahuangganhe*’ (D), were used in this study. Using conventional irrigation and fertilization strategies, all plum trees (six years old) grow under natural conditions. The climate of the experimental area is temperate and monsoonal with a mean annual temperature of 5.6 °C. The average annual precipitation is 594.8 mm, of which 80% falls from May to September. The fruit tissues were collected at the young fruit stage (abbreviated as Y, 18th May, 30 days after anthesis), color-change stage (abbreviated as C, 17th Jun, 60 days after anthesis) and maturation stage (abbreviated as D, 17th July, 90 days after anthesis). Three individuals were selected for each variety, and three fruits were randomly selected from one individual for each sampling stage, and then pooled as a repeat. 18 samples were collected (two varieties, three stages, three repetitions/stage/variety). For molecular analysis, tissue samples were directly snap-frozen in liquid nitrogen and kept at −80 °C.

### Fruit color characteristics

The fruit color characteristics of Ch84 and D were measured using a colorimeter (Colorimeter Ci7600, MI, DE, USA). L* value represents the lightness (+L: black, −L: white), a* (+a: red, −a: green), and b* (+b: yellow, −b: blue). The parameters of L*, a*, and b* were measured following the manufacturing instructions.

### RNA isolation and sequencing

The total RNA of these 18 pericarp samples was extracted using the TruSeq RNA Sample Preparation Kit (Autolab Biotechnology, Beijing) following the manufacturers guide. Then, the quality and quantity of the purified RNA were evaluated by 1% agarose gel, NanoDrop ND1000 spectrophotometer (NanoDrop Technologies, Wilmington, DE, USA) and Agilent 2100 Bioanalyzer (Santa Clara, CA, USA). The RNA-seq libraries were prepared using an Illumina TruSeq Stranded RNA kit (Illumina, San Diego, CA, USA). After enrichment of eukaryotic mRNAs with Poly A tail by magnetic beads with oligo (DT), then mRNAs was interrupting by a Ultrasonic Processor. Using the fragmented mRNAs as template, the first strand of cDNA was synthesized using random oligonucleotides as primers in M-MuLV reverse transcriptase system. Then, the RNA strand was degraded with RNaseH, and the second strand of cDNA was synthesized from dNTPs in DNA polymerase I system. The purified double stranded cDNA was repaired at the end, adding with a tail and connected to the sequencing connector. The cDNA of about 200 bp was screened with ampure XP beads, amplified by PCR, and the PCR product was purified again with ampure XP beads, and finally the library was obtained. Then, the prepared sequence libraries were sequenced on an Illumina nova 6000 sequencer under the pair-end 150 bp mode by Genedenovo Biotechnology Corporation (Guangdong, China).

### Transcriptome data analysis

The raw reads from transcriptome sequencing were filtered by FastQ (version 0.18.0) with the default parameters and the clean reads were assembled using Trinity (version 2.8.4). The power value of the samples was calculated using RNASeqPower software (V3.14) to evaluate whether the number of sample repetitions. Assembled contigs were annotated by BLASTx (Britt, 1992) against seven public databases: Nr, Nt, SwissProt, KOG, Pfam, GO, and KEGG with >90% identity and an *E*-value <0.00001. Unigene expression levels were calculated based on FPKM values. Then, differentially expressed genes (DEGs) among the sample groups were identified using the DESeq2 software (Love, Huber & Anders, 2014). DEGs were identified based on a false discovery rate (FDR) <0.05 and —log2 foldchange— ≥ 2. DEGs were then submitted to GO (http://www.geneontology.org/docs/go-enrichment-analysis/) and KEGG enrichment (http://www.kegg.jp/kegg) analysis to annotate their biological function and significantly metabolic pathways. The submission of DEGs to GO and KEGG enrichment was performed using GOseq (Young et al., 2010) and KOBAS 2.0 (Mao et al., 2005), respectively.

### RT-qPCR analysis

To analyze the expression of mRNA, RT-qPCR was performed with SYBR Green RT-qPCR master mix (TaKaRa, Japan) on an ABI7500 RT-qPCR system. The target genes of RT-qPCR were calculated with relative quantification (2^−ΔΔCt^) method, which was normalized to *Actin*.

### LC-MS and LC-MS/MS analysis

In order to study the difference in metabolites between the two cultivars at different developmental stages, untargeted metabolomics was carried out using LC-ESI-MS/MS system (Fang et al., 2019). We crushed freeze-dried fruit tissues in a mixer mill (MM 400, Retsch, Germany) to obtain the metabolites. Using 1.0 mL 70% aqueous methanol containing 0.1 mg/L lidocaine as the extraction solvent, 100 mg of sample powder was weighed and extracted overnight at 4 °C. Then, the mix was centrifugated 10,000 g for 10 min. In order to perform UPLC separation, two liters of sample solution were placed into an ACQUITY HSS T3 C18 column (100 × 2.1 mm, 1.7 µm; Waters) with a flow rate of 0.4 mL/min. The mobile phases were 0.04% acetic acid aqueous solution (A) and acetonitrile with 0.04% acetic acid (B). We separated the compounds following this gradient: 95:5 Phase A/Phase B at 0 min; 5:95 Phase A/Phase B at 11.0 min; 5:95 Phase A/Phase B at 12.0 min; 95:5 Phase A/Phase B at 12.1 min; 95:5 Phase A/Phase B at 15.0 min; 5:95 Phase A/Phase B at 12.0 min; 95:5 Phase A/Phase B at 12.1 min; 95:5 Phase A/Phase B at 15.0 min. An ESI-triple quadrupole-linear ion trap (Q TRAP)-MS was used to analyze the effluent. The operation parameters were listed in Table S1.

### Metabolomic data analysis

To produce a matrix containing fewer biased and redundant data, peaks were filtered to remove the redundant signals caused by different isotopes, in-source fragmentation, K+, Na+, and NH4+ adduct, and dimerization. Metabolites were quantified by comparison to the internal standard values and identified according to the internal database and public databases: MassBank (https://massbank.eu/MassBank/), KNApSAcK (http://www.knapsackfamily.com/KNApSAcK/), HMDB (https://hmdb.ca/), MoTo DB (Grennan & MoTo, 2009), and METLIN (Zhu et al., 2013). Orthogonal projection to latent structures-discriminant analysis (OPLS-DA) was applied to highlight significant biomarkers (Managa, Sultanbawa & Sivakumar, 2020). Variable importance of the projection (VIP) score of the application (O) PLS model was used to filter the best differentiated metabolites between groups. The threshold of VIP was set to 1. For univariate analysis, Student’s *T*-test with the Benjamini–Hochberg-based false discovery rate (FDR) was used to characterize the differential significances of metabolites among groups. Metabolites with FDR < 0.05 and VIP ≥ 1 were considered differential metabolites. Then, metabolites were mapped to KEGG metabolic pathways for pathway analysis and enrichment analysis.

## Results

### Phenotypic analysis of the two plum varieties

**Figure 1:**
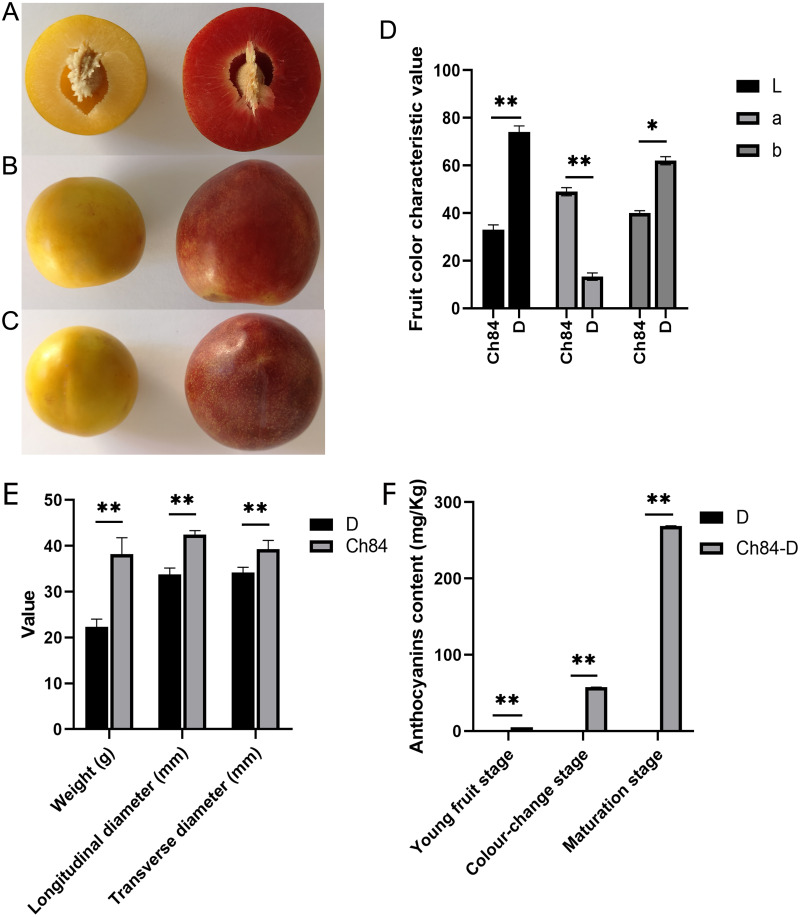
Fruit performance of the two plum varieties. (A–C) The color and size of the two varieties of plums at different angles. (D) The results of colorimeter analysis. L, a, and b represented brightness, red-green coordinates, and blue-yellow coordinates of samples, respectively. (E) The statistical results of transverse and longitudinal diameter and weight of two varieties of plum. (F) The content of anthocyanins in three different periods. Ch84 represents ‘*Changli84*’ (the red fruit); D represents ‘*Dahuangganhe*’ (the yellow fruit).

After 90 days of growth, plums gradually matured. There were obvious differences between the two varieties. Ch84 had a bigger fruit size than D. The fruit pericarp color of ch84 was red and D was yellow (Figs. 1A to 1C, Table S2). Also, the fruit color characteristics showed that D had higher brightness (L) and yellow-blue (b) value than Ch84, but lower red-green (a) value (Fig. 1D, Table S2). Also, the fruit weight, longitudinal diameter and transverse diameter of Ch84 were significantly higher than D (Fig. 1E). The most striking difference is the color of the fruit. As we know, anthocyanins are the pigments that give red, purple, and blue plants their rich coloring. In three different periods, the content of anthocyanins in Ch84 was significantly higher than that in D (Fig. 1F).

### Sequence data summary

All the libraries of the plum fruits produced 1,089,530,564 raw reads (150 bp length/read) with an average 60,529,476 reads per library. After filtering out low quality reads, 1,087,632,936 reads (99.83% of the raw data) were obtained. The Q20 and Q30 value was 97.59% and 93.07%, respectively. The quality of sequencing data was summarlied in Table S3. Trinity software assembled the high-quality reads into 37,151 transcripts with an average length of 1,216 bp and an N50 value of 2063. The range of transcripts length was 201–16,179 bp. The percentages of mapping to transcript were 89.69 and 87.37%, respectively. Moreover, we also evaluated the correlation between biological repeat samples by PCA and sample to sample correlation analysis. The results showed that samples in the same group were clustered together (Fig. S1A) and had high correlation coefficient (Fig. S1B), indicating that the difference between biological repeat samples was small. The statistical power, which was calculated by RNASeqPower, is 69.09%, 74.63% and 72.35% for the comparisons Ch84-Y *vs.* D-Y, Ch84-C *vs.* D-C, and Ch84-D *vs.* D-D, respectively (Table S4).

### Transcript’s annotation

All the assembled transcripts were BLAST-searched against six public databases (Nr, Nt, SwissProt, KOG, Pfam, GO and KEGG) using search tools. In total, 26,055 transcripts were annotated using these databases. The annotation summary was shown in Fig. S2A). There were 12,271 transcripts were commonly annotated in these databases (Fig. S2B). The species distribution with the greatest number of plum were *Prunus mume* (60.61%), *Prunus persica* (8.72%), *Malus domestica* (3.90%), *Pyrus x bretschneideri* (2.92%), *Populus trichocarpa* (2.86%), *etc* (Fig. S2C).

### Analysis of DEGs between the two plum varieties

**Figure 2:**
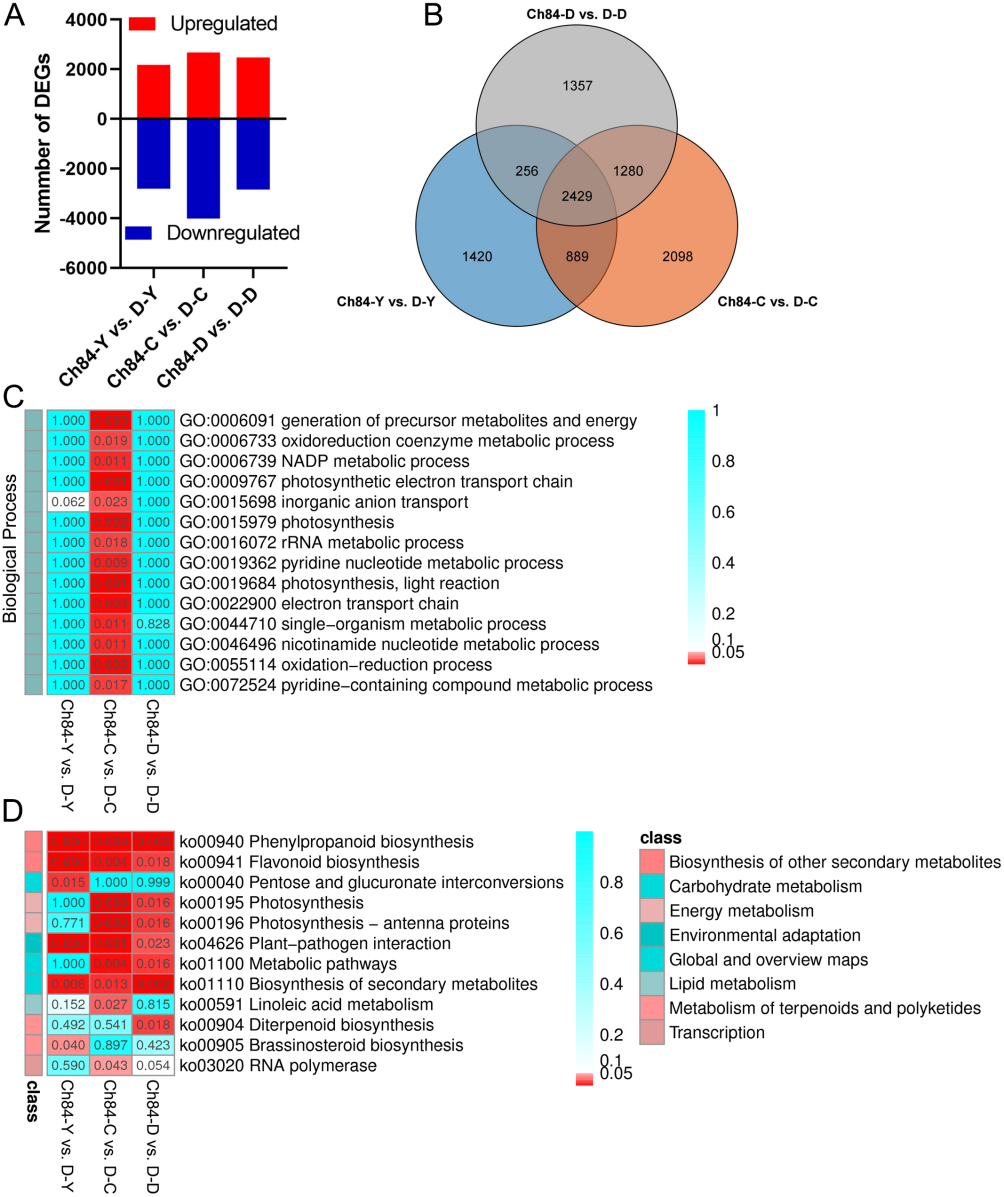
DEGs statistics between Ch84 and D and their functional annotation analysis. (A) The number of DEGs between the two plum varieties at different developmental stage. (B) The Venn analysis results of different comparisons. (C and D) GO and KEGG enrichment results of all the DEGs, respectivly. The redder the color, the higher the significance. Ch84 represents ‘*Changli84*’ (the red fruit); D represents ‘*Dahuangganhe*’ (the yellow fruit).

According to false discovery rate (FDR) <0.05 and —log2 foldchange— ≥ 2, DEGs were identified using the DESeq software package. As shown in Fig. 2A, 4,994, 6,696 and 5,322 DEGs were identified in the comparison of Ch84-Y *vs.* D-Y, Ch84-C *vs.* D-C, and Ch84-D *vs.* D-D, respectively (Table S5). Venn analysis shows that there are 2429 DEGs in common (Fig. 2B, Table S5). We conducted KEGG and GO functional enrichment analysis for all DEGs between the two varieties at the three developmental stages. GO enrichment was performed on all the DEGs to cluster the genes with similar functions.

The GO enrichment results showed that no significant BP terms enriched in Ch84-Y *vs.* D-Y and Ch84-D *vs.* D-D (Fig. 2C). For KEGG enrichment analysis, we found that the “Phenylpropanoid biosynthesis” (ko00940), “Flavonoid biosynthesis” (ko00941), “Plant-pathogen interaction” (ko04626) and “Biosynthesis of secondary metabolites” (ko01110) were significantly enriched (Fig. 2D). It is worth paying attention to the DEGs enriched in the “Flavonoid biosynthesis” pathway, including *F3H, CYP75A* (Flavonoid 3′, 5′-hydroxylase), *CHS, CHI, ANS, FLS, HCT, LAR, PGT1* and *DFR etc*. Also, four DEGs, CrtB, LCYB, LCY1 and LCYE, were significantly enriched in “Carotenoid biosynthesis” (ko00906) pathway (*P* > 0.05). Although not significant, this result reflects the enrichment of known fruit-related genes that interact with carotenoid biosynthesis. Hence, both “Flavonoid biosynthesis” and “Carotenoid biosynthesis” are important pathways that guiding us to reveal the mechanism of plum fruit color differences.

### DEGs in flavonoid biosynthesis process

**Figure 3:**
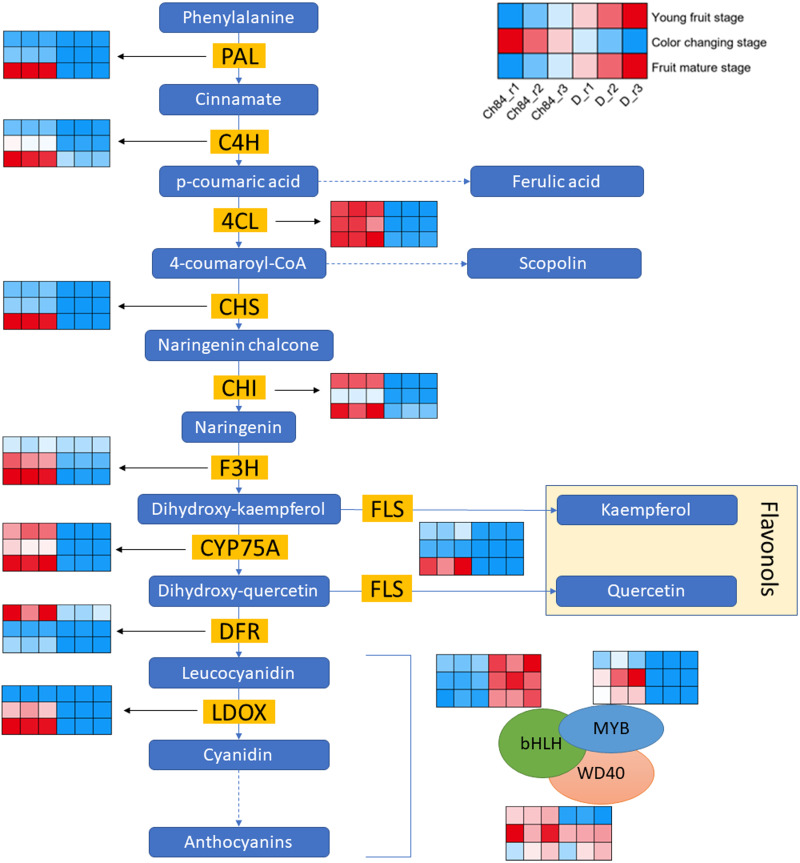
Expression pattern analysis of DEGs in flavonoid biosynthesis pathway between Ch84 and D. A simplified model depicting the transcription factors in flavonoid biosynthesis pathway. Red columns in the heatmap indicate higher gene expression, while blue columns in the heatmap indicate lower gene expression. Ch84 represents ‘*Changli84*’ (the red fruit); D represents ‘*Dahuangganhe*’ (the yellow fruit). The annotation of the heatmap cells were marked on the upper right corner.

Figure 3 shows the schematic of anthocyanin synthesis. Most of the key regulators had higher expression in Ch84 than that in D at the color-change stage and maturation stage, which corresponded to the color of the two plum fruits. Importantly, the *DFR,* one of the key genes in anthocyanin biosynthesis, had the highest level at the young fruit stage than other developmental stages. Overall, the activity of DEGs in flavonoid biosynthesis process in Ch84 was much higher than that in D.

In order to further explore the DEGs that related to fruit color, we identified the gene expression level of MBW protein complexes. We found that *MYB* and *bHLH* were significantly highly and lowly expressed in Ch84, respectively, at all the three stages. However, *WD40* showed significant difference at the young fruit stage, but not at the color-change and maturation stage.

### DEGs in carotenoid biosynthesis

**Figure 4:**
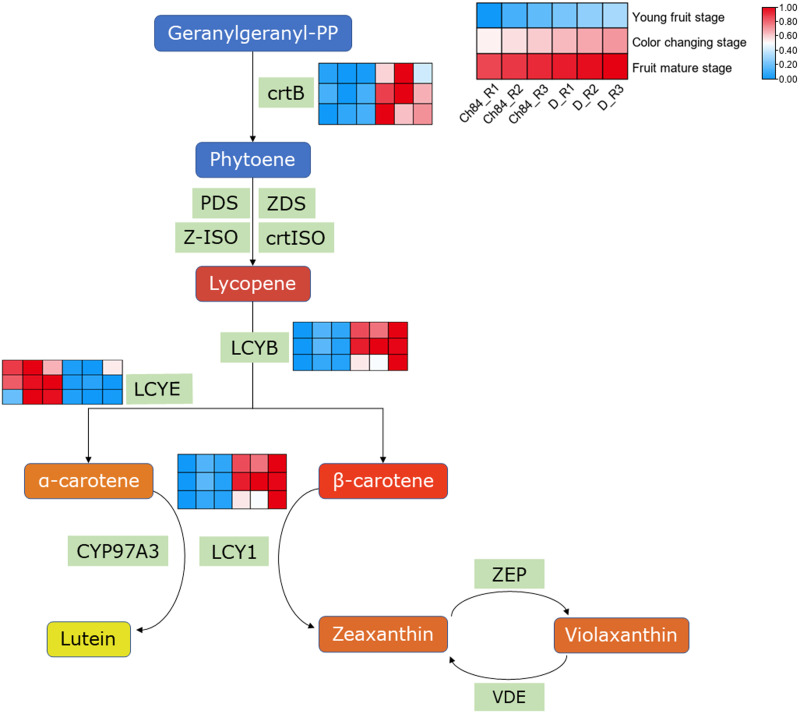
Expression pattern analysis of DEGs in carotenoid biosynthesis pathway. A simplified schematic depicting the DEGs in carotenoid biosynthesis pathway. The heatmap showed the expression of these DEGs. The redder the color, the higher the level. Ch84 represents ‘*Changli84*’ (the red fruit); D represents ‘*Dahuangganhe*’ (the yellow fruit). The annotation of the heatmap cells were marked on the upper right corner.

As we know, carotenes and carotenoids are the main substances that control fruit color. In carotenoid biosynthesis pathway, CrtB, LCYB and LCY1 had higher expression in D than that in Ch84 at all the three detected stages (Fig. 4). For LCYE, its expression was higher in Ch84 than in D (Fig. 4).

### qRT-PCR validation

**Figure 5:**
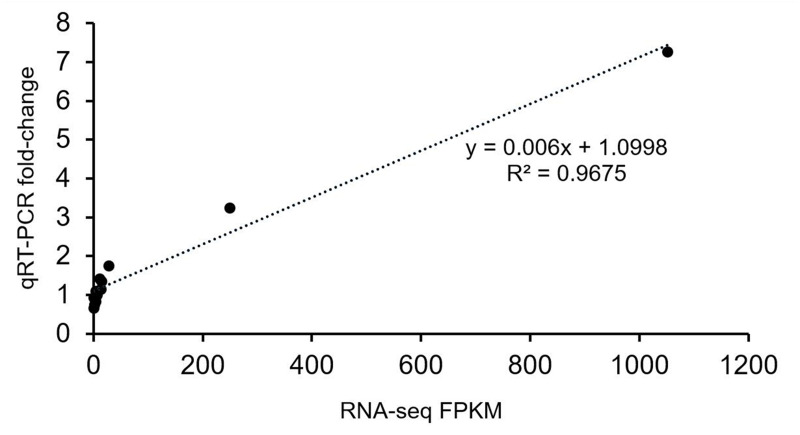
Verification analysis of candidate gene expression using qRT-PCR. Correlation analysis of candidate genes based on RNA-seq and qRT-PCR data. The abscissa indicates the FPKM value of transcriptome sequencing, and the ordinate indicates the fold-change value detected by qRT-PCR.

The gene expression profiles were validated by qRT-PCR. The results were closely consistent with the RNA-Seq results with a R^2^ value 0.9675 (Fig. 5). The discrepancies at different developmental stages between the qRT-PCR and RNA-Seq results might be caused by a sensitivity bias between the two methods.

### Analysis of differential metabolites (DMs) between the two plum varieties

**Figure 6:**
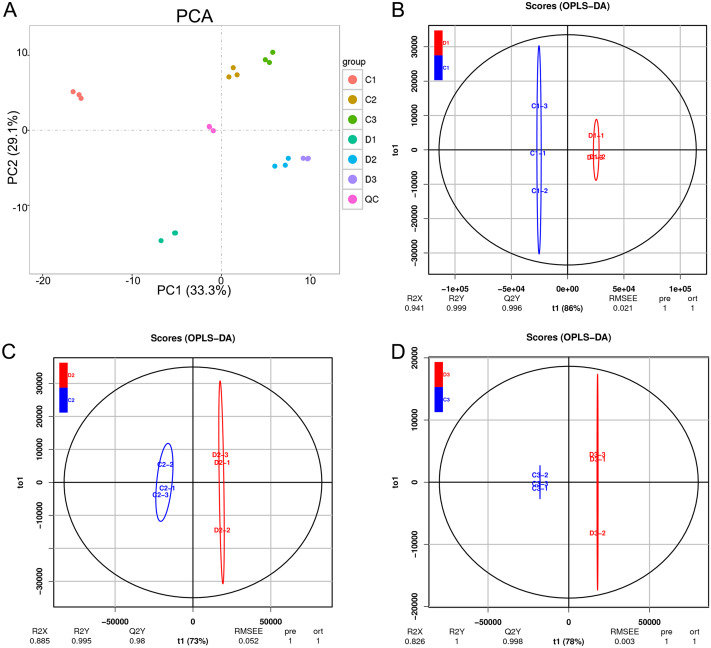
Summary of the metabolomic data. (A) The PCA results of metabolomic data of six sample groups; the colors represent different groups. (B, C, D) The OPLS-DA analysis results between the two plum varieties at the young fruit stage, color changing stage and fruit mature stage. Ch84 represents ‘*Changli84*’ (the red fruit); D represents ‘*Dahuangganhe*’ (the yellow fruit).

The unsupervised PCA was performed in both positive and negative spectra to distinguish the classes and assess the global metabolism variations. The PCA score plot of positive spectra indicated a clear classification of observations of Ch84 and D (Fig. 6A). To further distinguish the Ch84 and D and to identify differential variants, a supervised OPLS-DA was conducted. In Figs. 6B–6D, a remarkable separation of LC-MS data in Ch84 and D groups at different developmental stages was observed in the OPLS-DA score plot, indicating that this OPLS-DA model was non-overfitting (Table S2).

**Figure 7:**
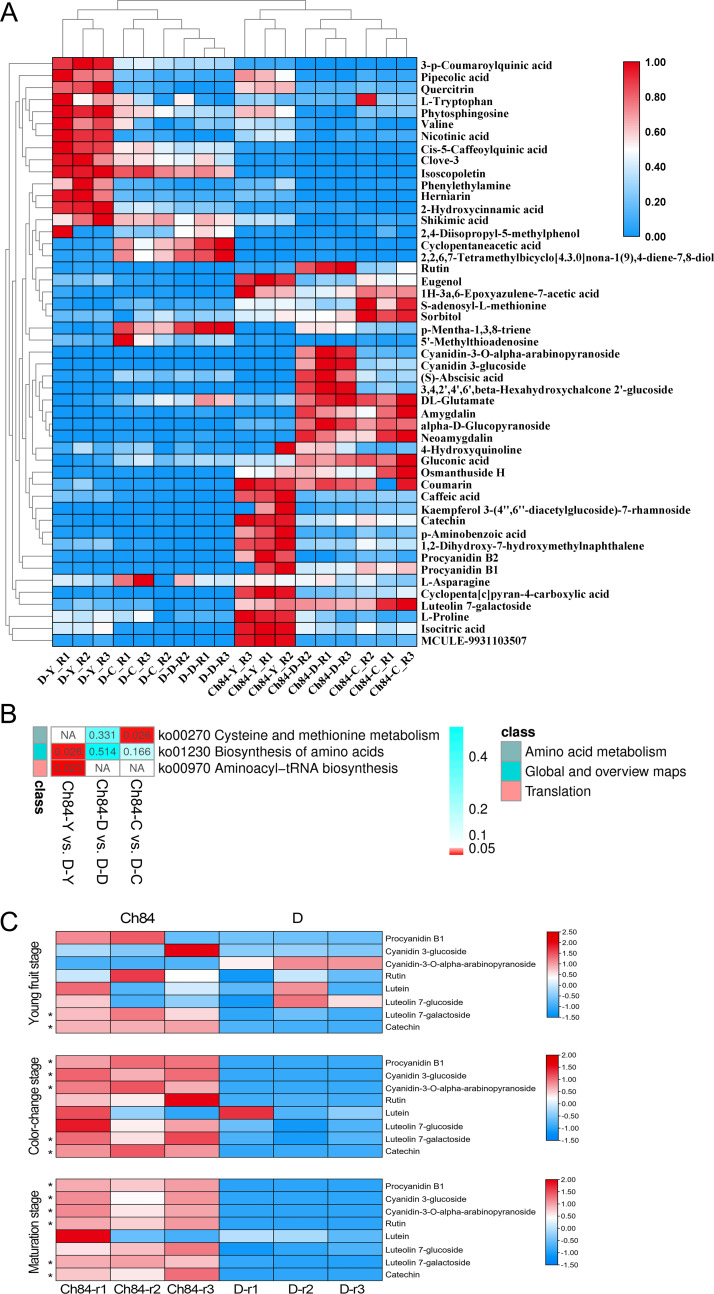
Differential metabolites and functional enrichment analysis. (A) The level of differential metabolites. The redder the color, the higher the level. (B) KEGG enrichment results based on these differential metabolites. (C) The level of pigment content metabolites of the two plums at different developmental stage. Ch84 represents ‘*Changli84*’ (the red fruit); D represents ‘*Dahuangganhe* ’ (the yellow fruit).

The results of these assessments mentioned above suggested that the LC-MS data quality was reliable. Fifty-four differential metabolites were identified and the heatmap of the differential metabolites were shown in Fig. 7A. The KEGG enrichment analysis was performed to uncover the most relevant biological pathways about fruit color. The KEGG enrichment and topology analysis demonstrated that main difference of metabolites between the two varieties was related to “Cysteine and methionine metabolism”, “Biosynthesis of amino acids” and “Aminoacyl-tRNA biosynthesis” at the metabolism level (Fig. 7B). We were further intrigued to see that, specifically with regard to fruit color, the pigment content was showed in Fig. 7C. In total, six pigment related metabolisms were detected in the LC-MS analysis. Four of the six showed significant differences between Ch84 and D, but not at each developmental stage. The content of Procyanidin B1, Cyanidin 3-glucoside, Cyanidin-3-O-alpha-arabinopyranoside, Luteolin 7-galactoside and Catechin were significantly higher in Ch84 than that in D at both color-change stage and fruit mature stage. Rutin showed significantly difference between Ch84 and D only at the fruit mature stage. However, there was no significant difference found in the level of Lutein and Carotenoids.

## Discussion

There are obvious differences in fruit color between the two plum varieties. Finding out the difference can help us understand the mechanism of fruit color. As shown in Fig. 1, The fruit color of Ch84 is dark red, while that of D is yellow. The main reason for this difference may be caused by different anthocyanin content in the plum fruit. The transcriptome analysis results showed that “Flavonoid biosynthesis” was significantly enriched in the KEGG analysis. Flavonoid biosynthesis is one of the most extensively studied secondary metabolic pathways in plants (Winkel-Shirley, 2001; Grotewold, 2006). Flavonoid biosynthesis is regulated by a complex network of signals triggered by internal metabolic cues and external signals, including visible light, pathogen attack, nitrogen, phosphorus, and iron deficiencies, temperature, *etc* (Abeynayake et al., 2012). Genetic characteristics are also a factor that cannot be ignored. The results of flavonoid biosynthesis related gene expression showed that their expression in the two plum cultivars were quite different. In particular, the expression trend of some DEGs was opposite between the two cultivars, such as *CHS*, *DFR* and *FLS*. Anthocyanin synthesis is a process that involves many steps (Lancaster & Dougall, 1992; Pesch et al., 2015; Gonzalez et al., 2008). *PAL, CHI, CHS, F3H, DFR*, *ANS* and *UFGT* are closely related with anthocyanin biosynthesis (Grotewold, 2006; Allan, Hellens & Laing, 2008). *CHS* is responsible for initiating flavonoid biosynthesis (Austin & Noel, 2003) and Flavonoid-3-O Glycosyltransferases (F3GT) has been shown to be responsible for anthocyanin biosynthesis (Montefiori et al., 2011). Peng reported that *CHS* and *F3GT* are crucial for total anthocyanin accumulation (Peng et al., 2019). In this present study, most of the genes in the flavonoid biosynthesis process had higher expression level in Ch84 than that in D, especially at the color-change and maturation stage, indicating that anthocyanins accumulated rapidly in these two periods in Ch84. Meanwhile, the metabolomic data also support this theory, because Procyanidin B1, Cyanidin 3-glucoside and Cyanidin-3-O-alpha-arabinopyranoside showed a higher abundance in Ch84 than D at these two stages. Interestingly, the *DFR* at the young fruit stage had the highest level, indicating the accumulation of catechin at the early development stage. The content of catechin in metabolome analysis was consistent with *DFR* level. *DFR* was identified as the main regulatory element of the catechin biosynthesis pathway (Kausar et al., 2020). In some engineered bacteria, the optimal production of anthocyanins required catechin (Zhang et al., 2022), but we do not know whether it is applicable to plum. Further experiments are needed to prove this. The expression of *CHS* increased with the growth of fruit in Ch84, but decreased in D, indicating that the anthocyanins in Ch84 were accumulated continuously, while those in D were consumed. Correspondingly, the results of LC-MS data also confirmed that anthocyanin content changed from procyanidin to cyanidin. In addition, *CHI* maintained a high level in Ch84, but decreased in D. In general, the flavonoid pathway begins with the sequential condensation of one molecule of 4-coumoryl-CoA and three molecules of malonylCoA by *CHS*, resulting in the formation of naringenin chalcones (Dao, Linthorst & Verpoorte, 2011). The chalcone is then stereospecifically isomerised to the flavanone naringenin by the enzyme CHI (Kimura, Aoki & S-I, 2001). Hence, the expression of *CHI* also plays an important role in the accumulation of anthocyanin. According to our results, the activity of flavonoid biosynthesis is determined by varieties, and the high expression of key genes leads to the changes of fruit color.

It is usually considered that anthocyanin biosynthesis is regulated by MBW complexes consisting of different MYBs, bHLHs and WD40 transcription factors (Liu et al., 2018). But there are exceptions. Myb and bHLH transcription factors can also co-regulate the expression of anthocyanin biosynthesis genes without depending on WD40 protein (Gao, 2020). The results of this study suggested that the differential expression of *MYB* and *bHLH* are key factors leading to fruit color change by regulating the activity of MBW, but there may not be a significant correlation between *MYB* and *bHLH* in our results. Combined with metabolomics, we further confirmed the role of anthocyanins in the color difference of plum fruits. We believe that the overall expression levels of genes in flavonoid biosynthesis process dominate the color change of plum fruit. The role of WD40 is limited.

The metabolomics results also give us a lot of hints about the mechanism of plum fruit color. Polyphenols including yellow flavonoids, procyanidins (B1 and B2) and cyanidin-3-O-glucoside in substantial amounts have been characterized in different palm fruits (Agostini-CostaTDS, 2018). In our results, procyanidin B1 and B2 had the highest level at young fruit stage in Ch84 and the content of procyanidin B2 decreased sharply at the color change stage. Conversely, the content of cyanidin increased with the growth of fruit and reached the peak at the maturation stage. While for D, the metabolites mentioned above did not change significantly at all developmental stages. As we know, procyanidins are members of the proanthocyanidin (or condensed tannins) class of flavonoids. They are oligomeric compounds, formed from catechin and epicatechin molecules. They yield cyanidin when depolymerized under oxidative conditions (Rue, Rush & van Breemen, 2018). Therefore, we speculated that the content of polyphenols, like procyanidin B1 and B2, in plums might be the leading factors of the matured fruit color.

For the carotenoid biosynthesis process, we found some genes were differently expressed, such as *CrtB, LCYB, LCY1* and *LCYE*. But neither carotenes nor carotenoids showed significant differences between Ch84 and D, indicating that carotene and carotenoids are not the dominate cause of plum fruit color difference.

## Conclusion

The level of genes and metabolites in the “flavonoid biosynthesis” pathway indicated that anthocyanins content is the dominate factor for the difference of fruit color between the two varieties. DEGs such as *CHS, CHI, DFR, CYPs, MYB* and *WD40* were the key regulators. Metabolites such as procyanidin B1, cyanidin 3-glucoside and cyanidin-3-O-alpha-arabinopyranoside were the key pigment (or precursor). While carotene and carotenoids were not shown to be key regulations with fruit color.

##  Supplemental Information

10.7717/peerj.14413/supp-1 Supplemental Information 1 Correlation between biological repeat samples by PCA and sample to sample correlation analysis A, the geometry indicates the samples of different groups. B, heatmap of the inter-individual correlation of all mRNA transcripts. Ch84 is short for *Changli84*; D is short for *Dahuangganhe*.

10.7717/peerj.14413/supp-2 Supplemental Information 2 The transcripts annotation summary A shows the number of transcripts in different annotation databases. B shows the Venn analysis results of the annotation results. Different color respects different database. C shows the species annotation information, the more circles there are, the higher the proportion is. Different colors indicate different species.

10.7717/peerj.14413/supp-3 Supplemental Information 3 Operation parameters

10.7717/peerj.14413/supp-4 Supplemental Information 4 Raw data of Figs. 1D, 1E, 1F and Fig. 6

10.7717/peerj.14413/supp-5 Supplemental Information 5 Overview of sequencing data

10.7717/peerj.14413/supp-6 Supplemental Information 6 Power analysis between different groups

10.7717/peerj.14413/supp-7 Supplemental Information 7 Information of all differentially expressed genes (DEGs) and the Venn analysis results The first three sheets in the table represent the DEGs of each comparision It contains the information of gene expression level and KEGG & GO annotation The last sheet shows the results of Veen analysis

## Additional Information and Declarations

### Data Availability

The following information was supplied regarding data availability: The original transcriptome sequencing data is available at NCBI: PRJNA726302. The Prunus salicina transcriptome shotgun assembly (TSA) is available at NCBI: GJEK00000000.
