# Supplementary figures and images for: Transcriptome and metabolome analyses reveal anthocyanins pathways associated with fruit color changes in plum (Prunus salicina Lindl.)
Source: PeerJ. 2022 Dec 13;10:e14413. doi: 10.7717/peerj.14413 (PMC9756864; doi:10.7717/peerj.14413)

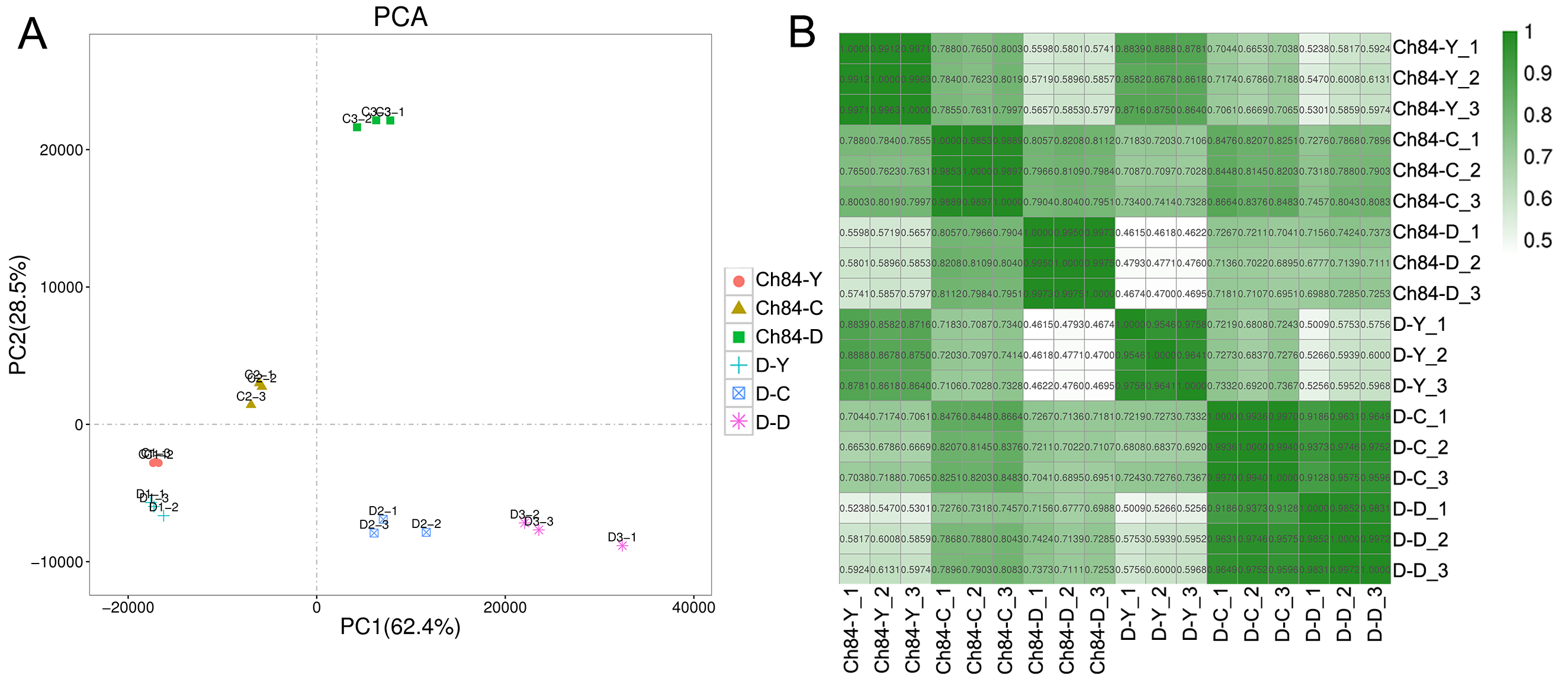

Supplement: Supplemental Information 1 — A, the geometry indicates the samples of different groups. B, heatmap of the inter-individual correlation of all mRNA transcripts. Ch84 is short for Changli84; D is short for Dahuangganhe. [file peerj-10-14413-s001.png]

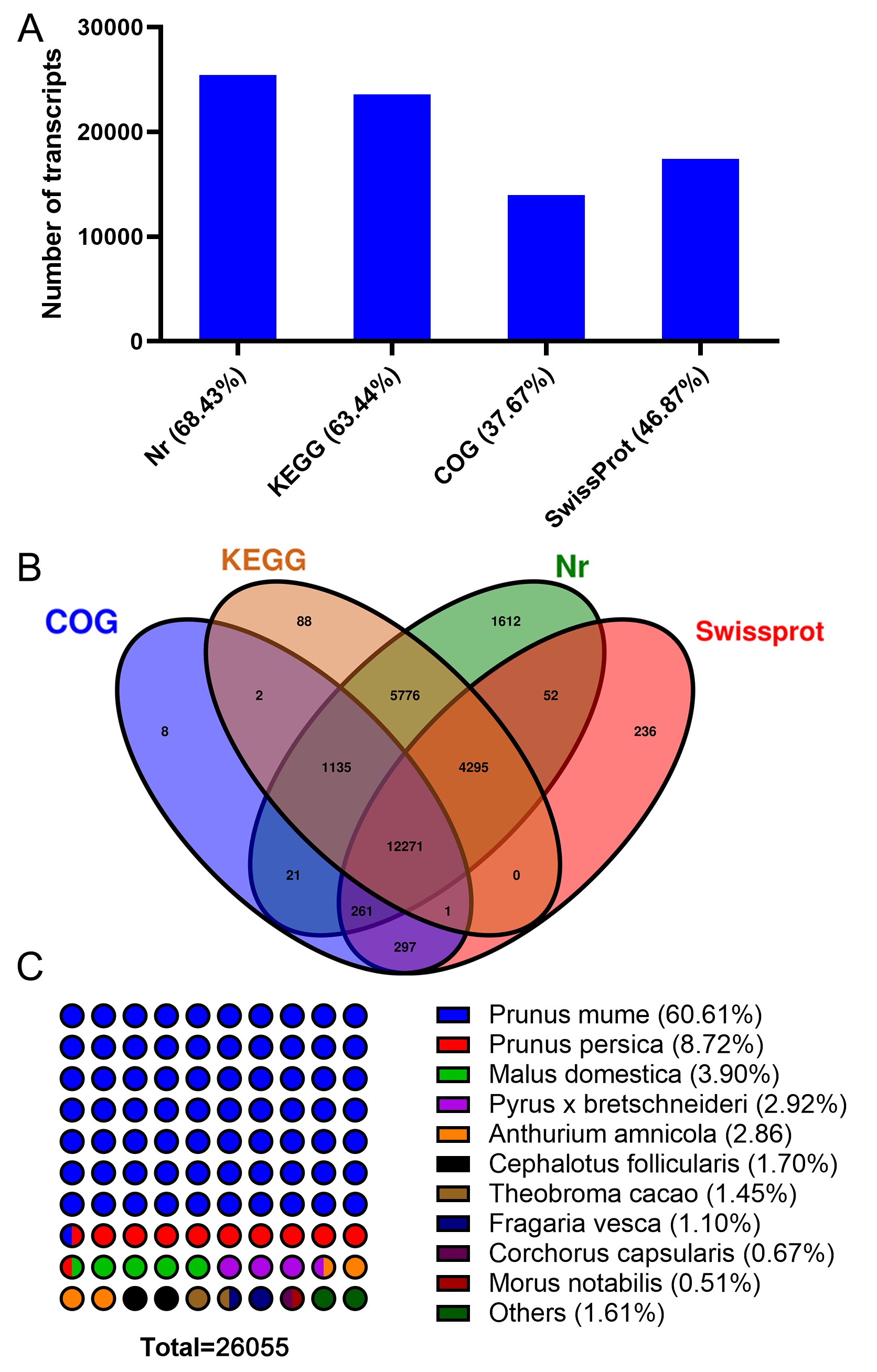

Supplement: Supplemental Information 2 — A shows the number of transcripts in different annotation databases. B shows the Venn analysis results of the annotation results. Different color respects different database. C shows the species annotation information, the more circles there are, the higher the proportion is. Different colors indicate different species. [file peerj-10-14413-s002.jpeg]
